# Supplementary material for: Uncovering near-free platinum single-atom dynamics during electrochemical hydrogen evolution reaction
Source: Nat Commun. 2020 Feb 25;11:1029. doi: 10.1038/s41467-020-14848-2 (PMC7042219; doi:10.1038/s41467-020-14848-2)
Supplement: Supplementary file 2 — Supplementary Information [file 41467_2020_14848_MOESM2_ESM.pdf]

Supplementary Information

Uncovering near-free platinum single-atom dynamics during electrochemical hydrogen evolution reaction

Fang et al.

Supplementary Figures

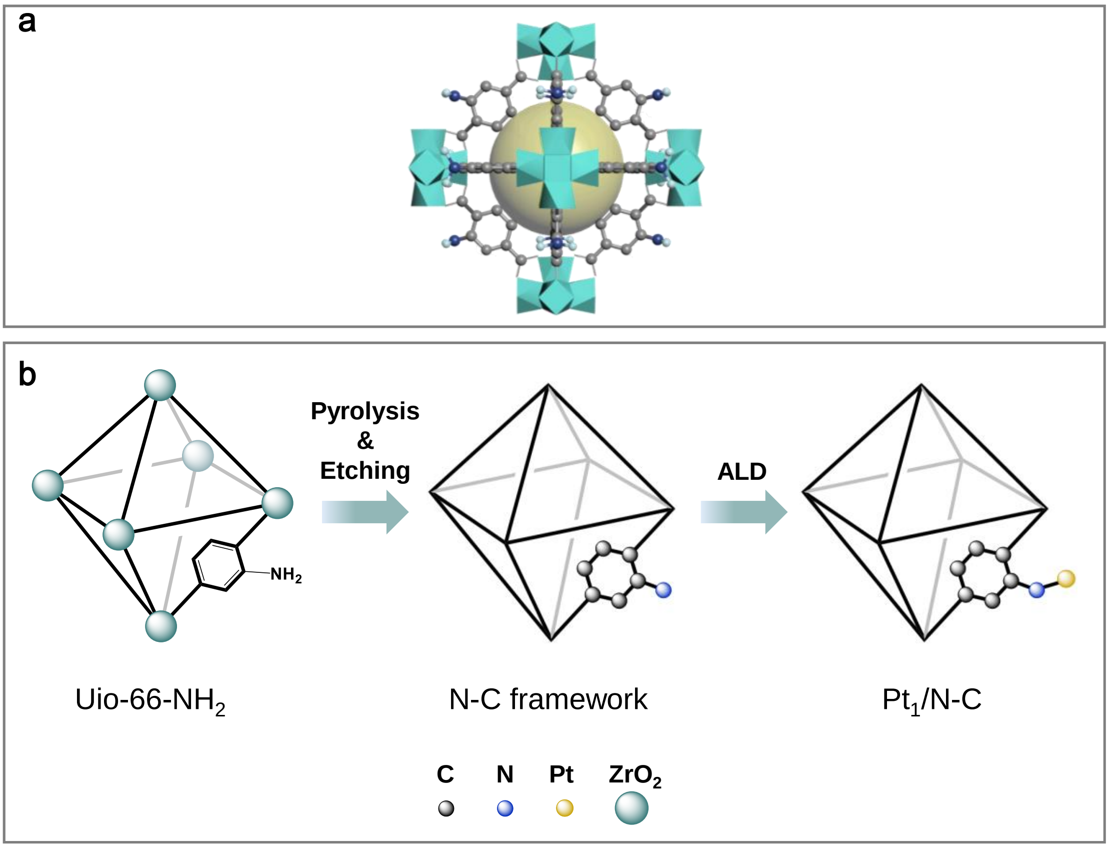

Supplementary Figure 1. Schematic illustration of the Uio-66-NH_2 structure (a) and synthetic mechanism for Pt_1/N-C (b).

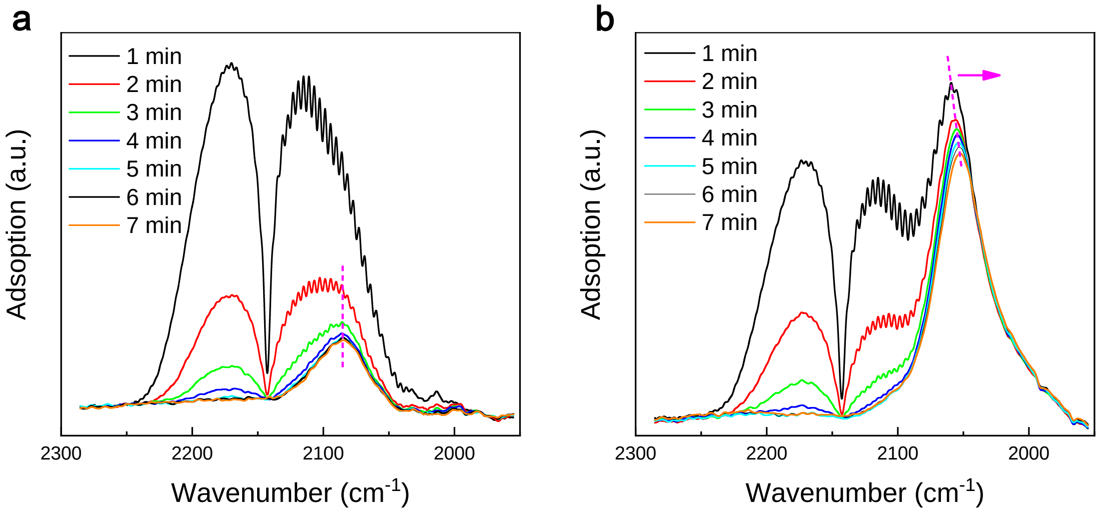

Supplementary Figure 2. CO-DRIFTS spectra of the as-prepared single atomic Pt_1/N-C sample (a), and the Pt nanoparticles (Pt-NPs) sample (b). The band at ~2084cm^{-1} in (a) remain unchanged during Ar purging, resulting from CO strongly adsorbed on Pt single atoms. While the band at ~2058 cm^{-1} in (b) shift to ~2052 cm^{-1} after Ar purging, indicating CO adsorption on Pt nanoparticles.

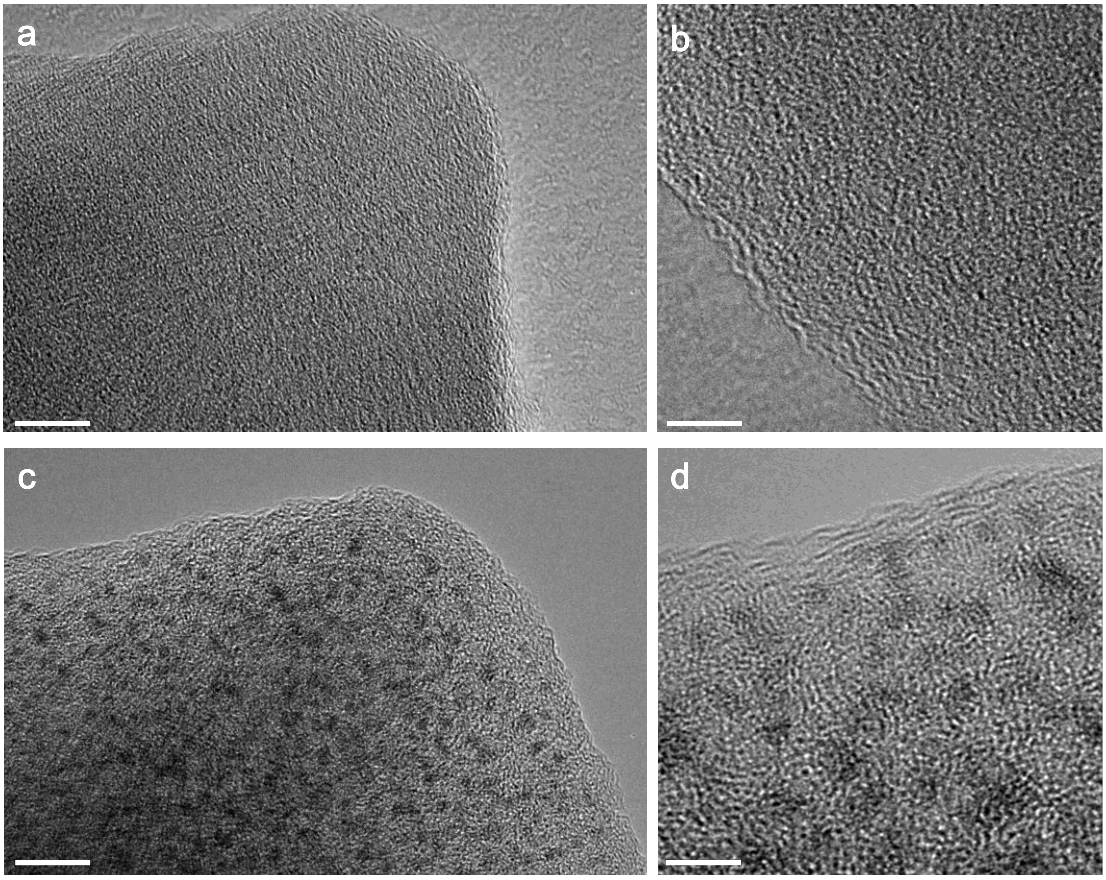

Supplementary Figure 3. High-resolution TEM images of the single atomic Pt_1/N-C sample (a), (b) and the Pt-NPs sample (c), (d). In (a) and (b), no obvious Pt-related clusters/nanoparticles were observed. After H_2 treatment (300 ℃, 2h), nanoparticles with ~2 nm can be seen in (c) and (d). Lengths of scale bars are 10 nm for (a) and (c), 5 nm for (b) and (d), respectively.

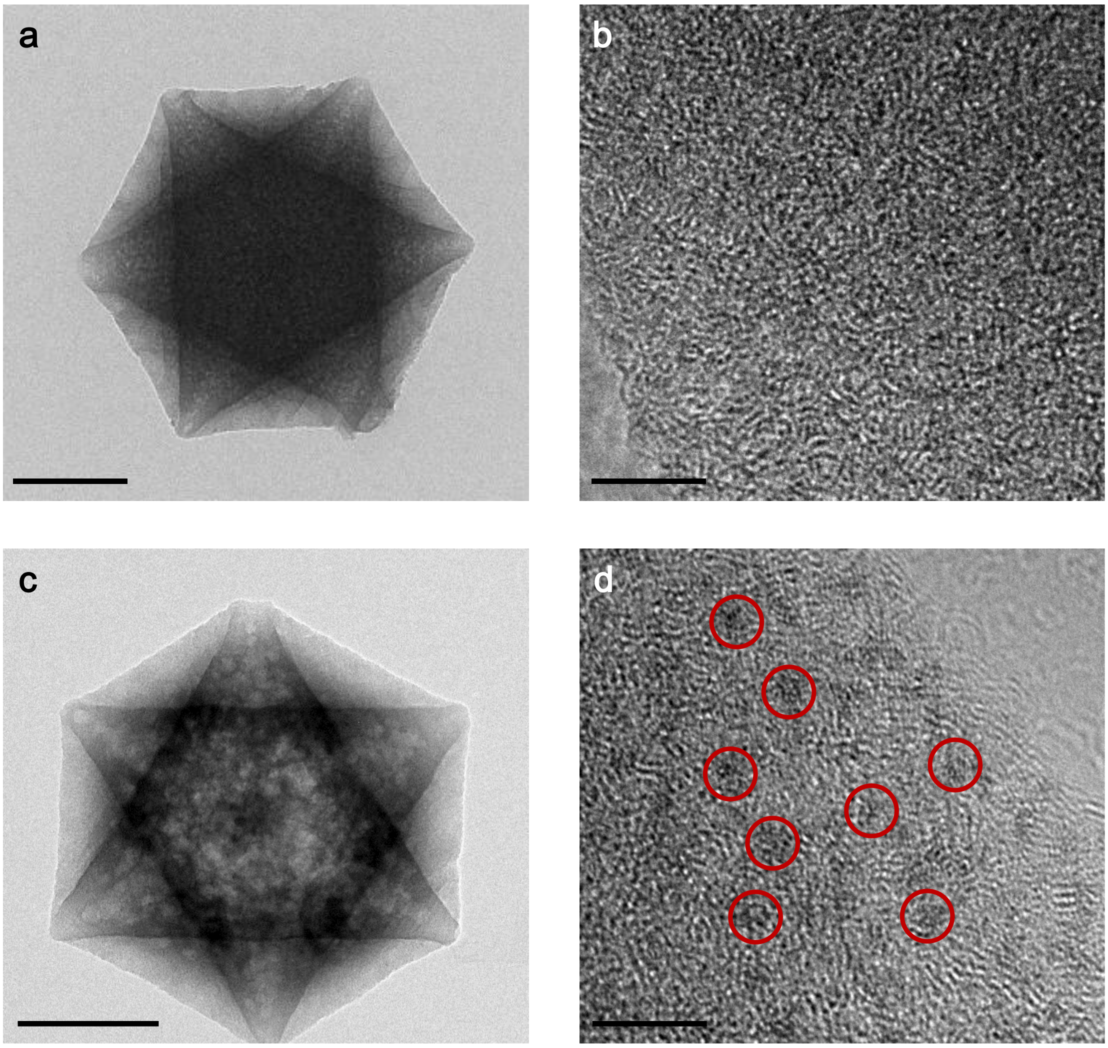

Supplementary Figure 4. Low magnification TEM images of Pt ALD on Uio66-NH_2 derived N-C framework (a) and on Uio66 derived C framework (c), and the corresponding high magnification TEM images in (b) and (d), respectively. Obvious clusters of Pt on the C framework can be discerned. The uncoordinated N atoms in the N-C framework play an important role in immobilizing Pt atoms avoiding from aggregating. Lengths of scale bars are 200 nm for (a) and (c), 5 nm for (b) and (d), respectively.

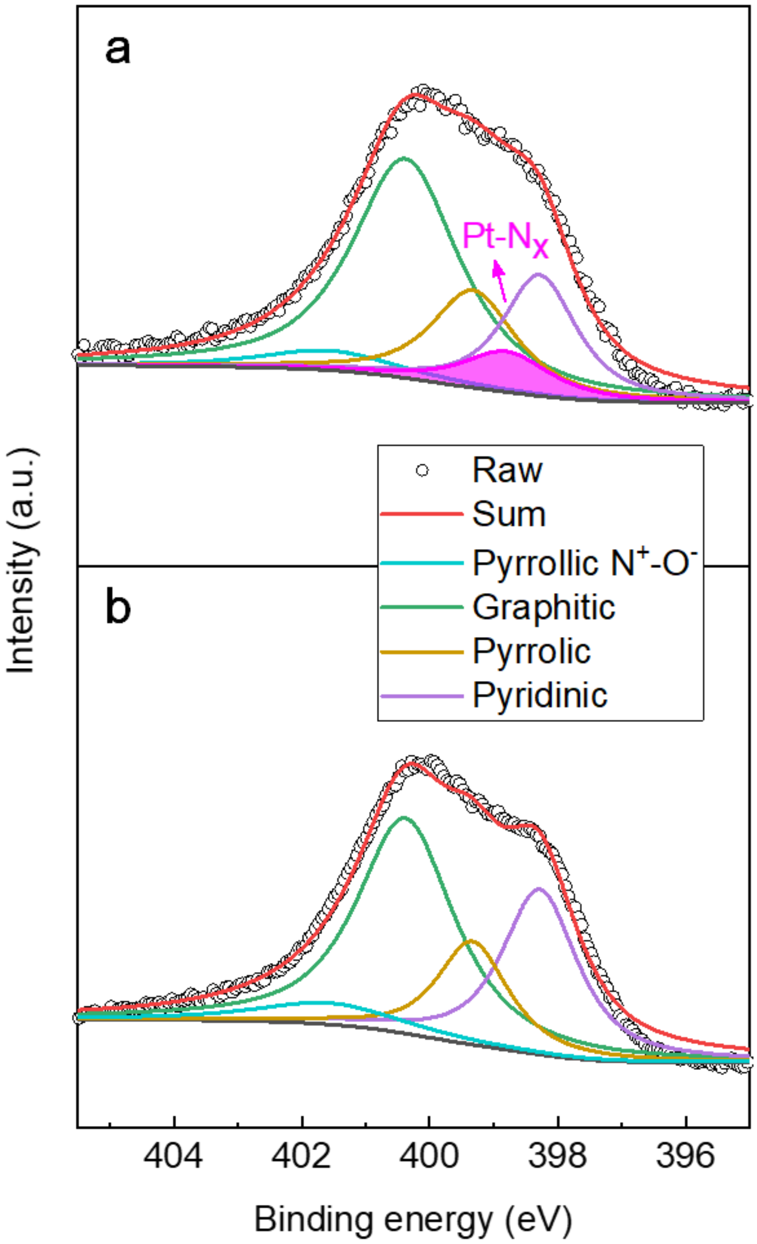

Supplementary Figure 5. XPS spectra of the Pt_1/N-C sample (a) and the pristine NC substrate (b). After Pt deposited on the N-C substrate, a novel peak appears between the pyrrolic and pyridinic peak can be assigned to Pt-Nx species.

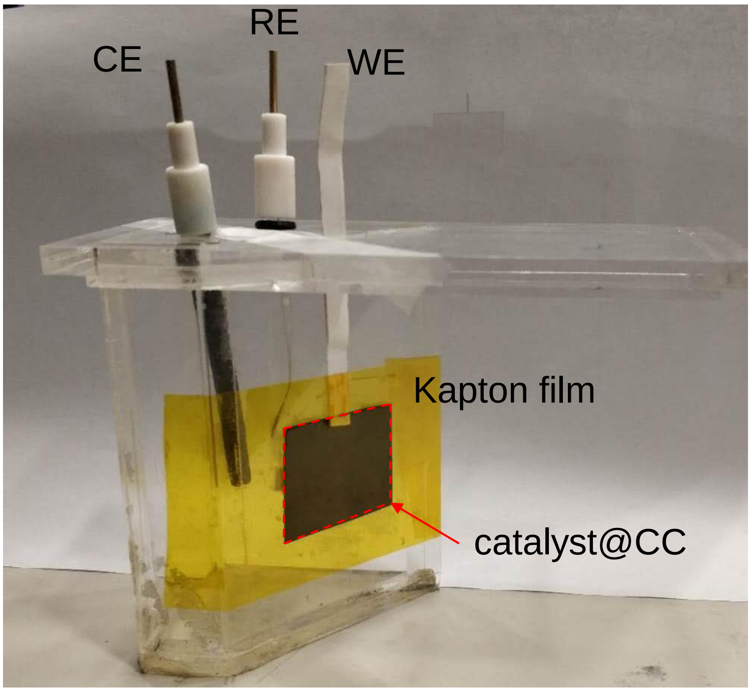

Supplementary Figure 6. The electrochemical cell for operando XAFS measurement. The catalysts were loaded on the carbon cloth (catalyst@CC) as working electrode (WE), led by a piece of copper tape to connect the electrochemical station. Ag/AgCl and carbon rod were used for the reference electrode (RE) and counter electrode, respectively. On the back of the carbon cloth, Kapton film was used to seal the cell flank.

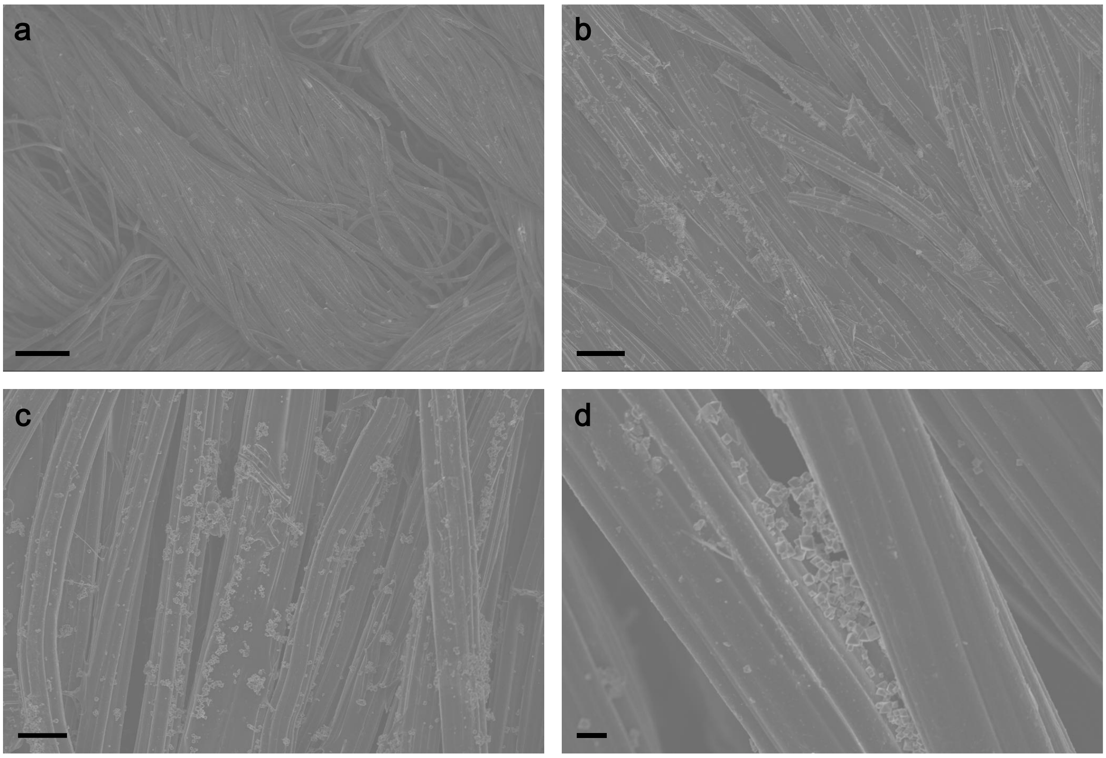

Supplementary Figure 7. SEM images of the carbon cloth coated with the catalysts in different magnifications. The catalysts were homogeneously filled in the gap of the carbon cloth, ensuring good contact between catalysts and electrolyte, also avoiding the catalysts from exfoliation during the measurement. Lengths of scale bars are 100, 20, 10 and 2 μm for (a), (b), (c) and (d), respectively.

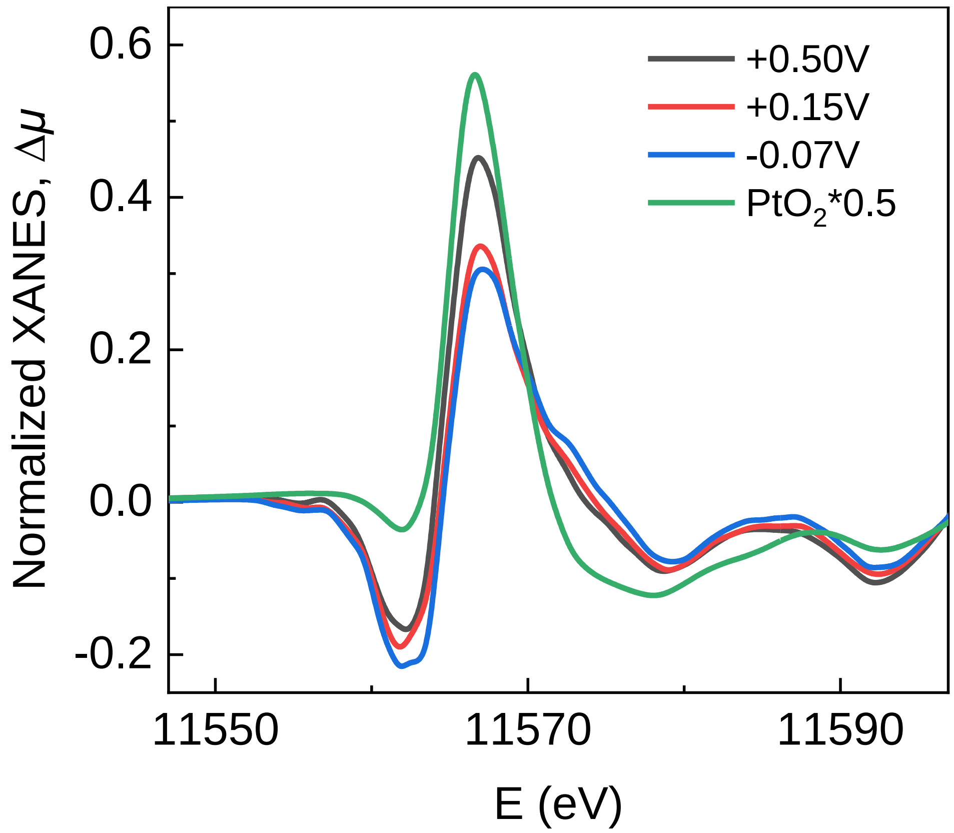

Supplementary Figure 8. Normalized difference spectra for Pt L_3-edge XANES using Pt foil as reference. The oxidation states and the *d*-band hole counts are fitted through integrating the area of the white-line peak from 11562.0 to 11578.8 eV.

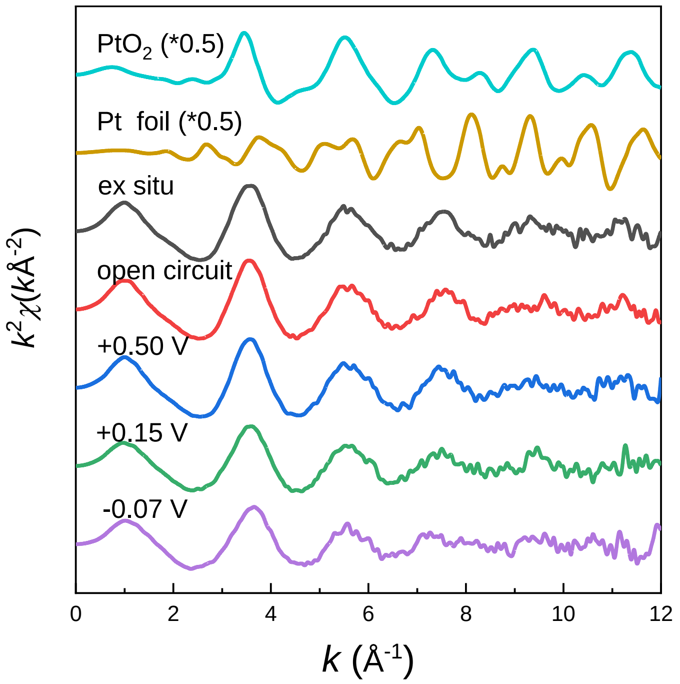

Supplementary Figure 9. The $k^2\chi(k)$ oscillations of Pt L_3-edge operando EXAFS analysis for the sample in different conditions, with the data of PtO_2 and Pt foil as references.

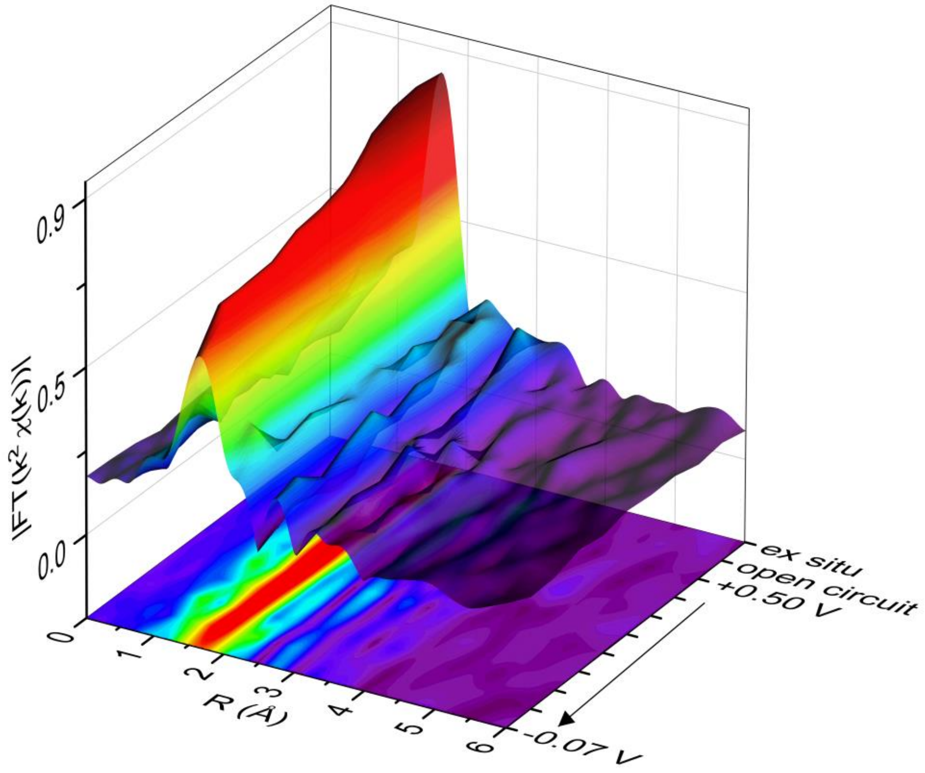

Supplementary Figure 10. Three-dimensional profile plot of successive operando Pt L_3-edge EXAFS spectra acquired in different working conditions, in corresponding to the XANES spectra in Fig. 1d.

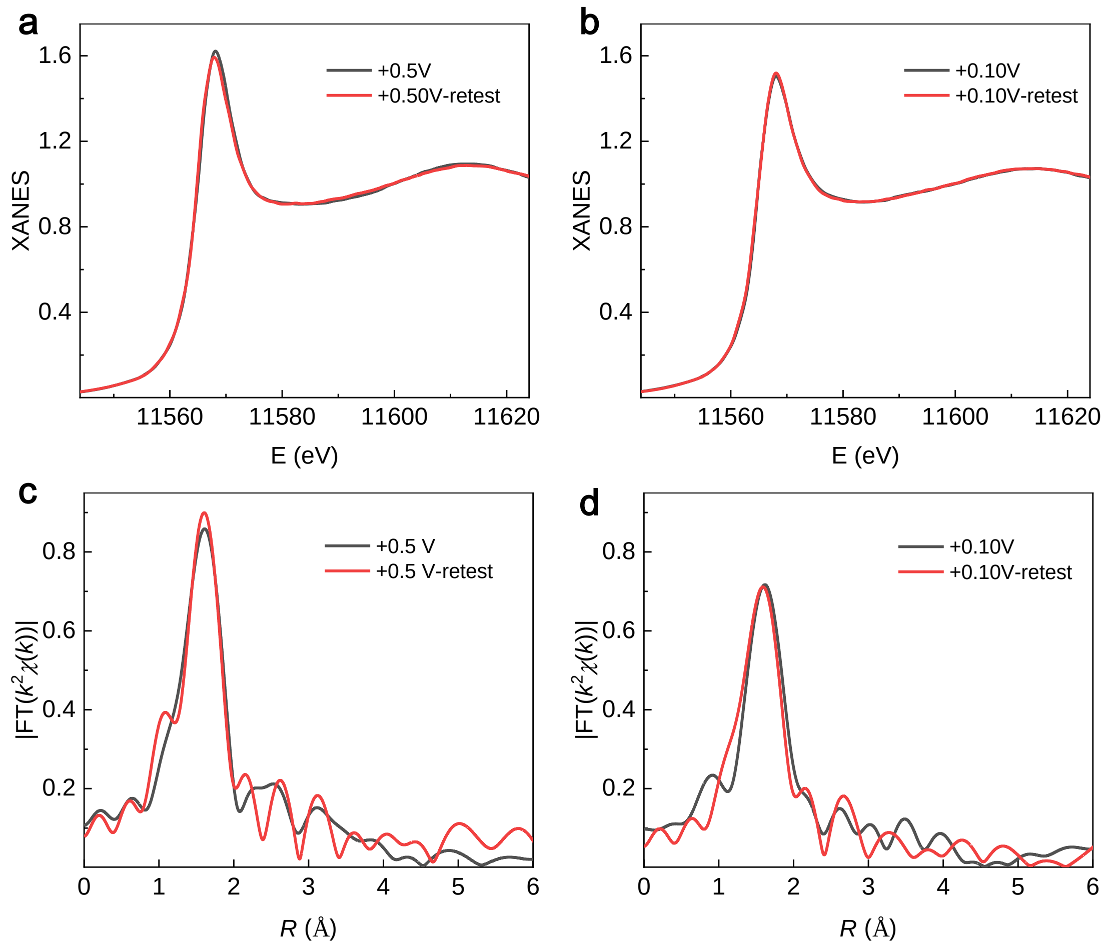

Supplementary Figure 11. The reversibility tests under +0.50 V (a, c) and +0.10 V (b, d). During the operando XAFS measurement, the working potential was set from +0.50 V to -0.07 V in sequence. After that, the electrode was naturally dried in air, then reinstalled to the cell and retest under +0.50 V and +0.10 V.

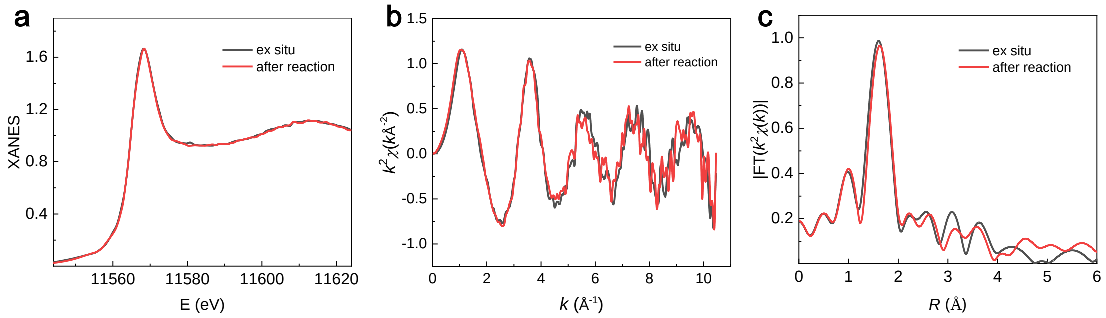

Supplementary Figure 12. The Pt L_3-edge XANES (a), $k^2\chi(k)$ oscillations (b), EXAFS (c) spectra of the samples in ex-situ and after reaction conditions. The sample after reaction derives from electrocatalyzing (at an overpotential of 100mV) for an hour in 0.1 M NaOH and then naturally dried in air. The strong consistency in these curves of ex-situ and after reaction demonstrates that the evolutions of Pt sites are well reversible.

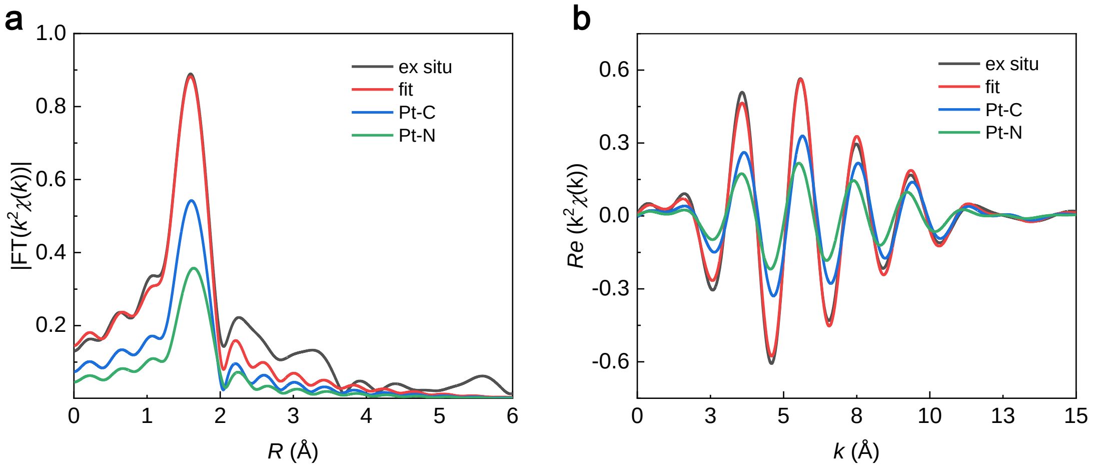

Supplementary Figure 13. First-shell fitting of EXAFS spectra (a), and corresponding Re($k^2\chi(k)$) oscillations (b) of two fitting paths (Pt-C and Pt-N) for the ex-situ sample. Black and red lines represent measured and fitted curves, respectively.

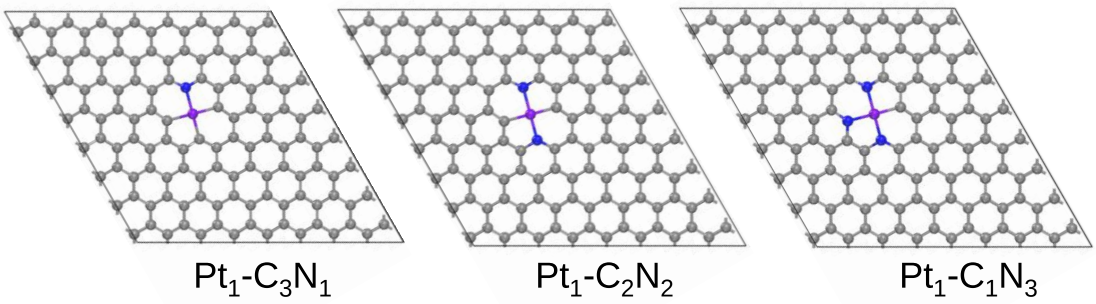

Supplementary Figure 14. Atomic configurations of the models of Pt_1-C_3N_1, Pt_1-C_2N_2 and Pt_1-C_1N_3, bader charge analysis was conducted based on these configurations to verify the geometric structure of the ex-situ state.

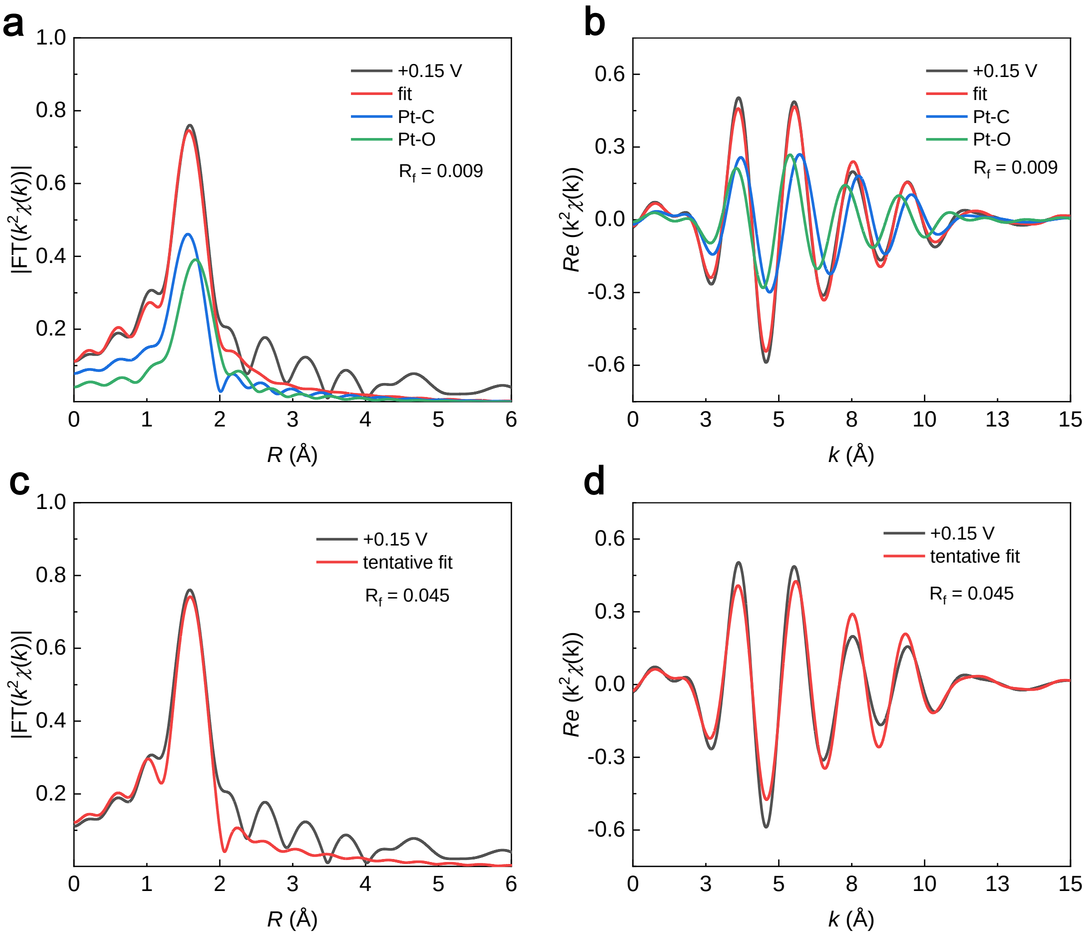

Supplementary Figure 15. First-shell fitting of EXAFS spectra (a) and corresponding Re ($k^2\chi(k)$) oscillations (b) of two fitting paths (Pt-C and Pt-O) for the +0.15 V sample. (c) and (d) show the tentative fitting of Pt-C_3 configuration, of which the R-factor is much larger than that of the Pt-C_2O_1 configuration.

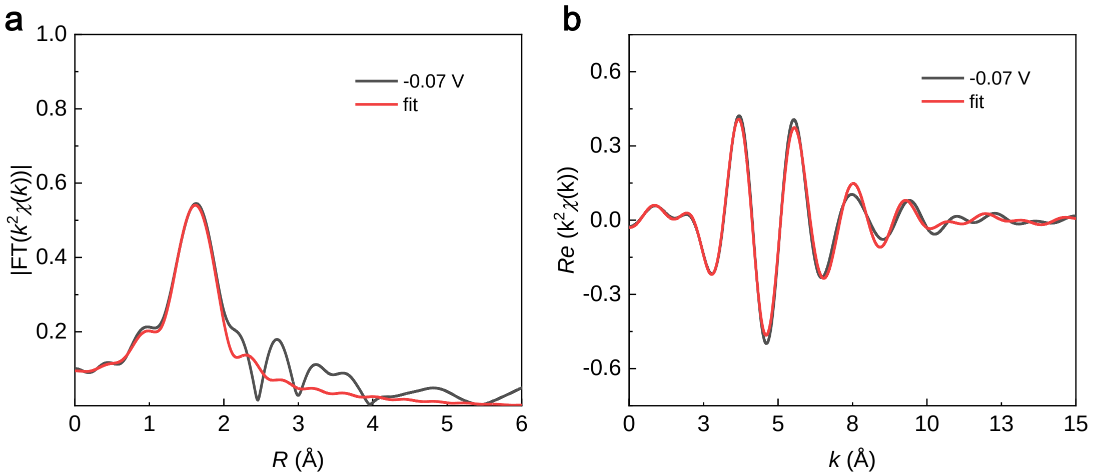

Supplementary Figure 16. First-shell fitting of EXAFS spectra (a), and corresponding Re($k^2\chi(k)$) oscillations (b) for the -0.07 V sample, only the Pt-C path was considered in this fitting.

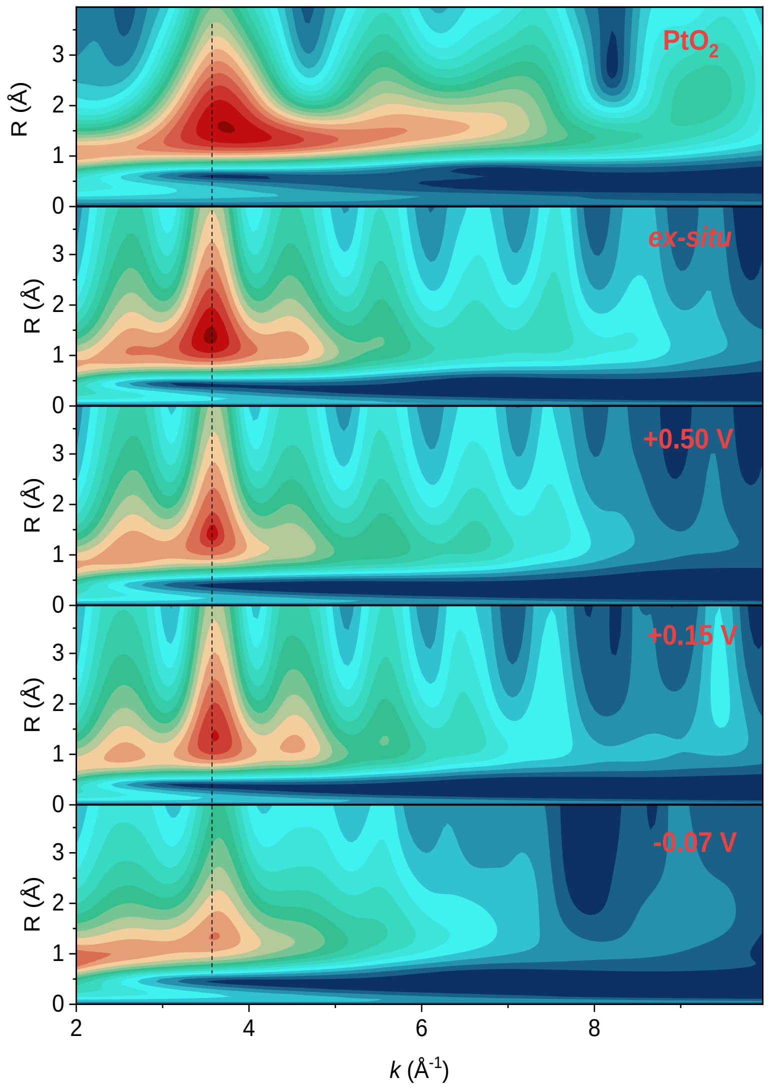

Supplementary Figure 17. Wavelet transform contour spectra for the k^2-weighted EXAFS data of different conditions. For the PtO_2 sample, the WT intensity maximum near 3.73 Å^{-1} can be attributed to the Pt-O coordination. For the ex-situ sample, the WT intensity maximum locates at ~ 3.55 Å^{-1}, a little lower K than that of PtO_2, indicating that Pt coordinates with lighter atoms than O, i.e. C and/or N atoms. For the +0.50 V sample, the position of the WT center remain unchanged, consistent with the previous EXAFS analysis in the main text. Interestingly, the WT center of + 0.15 V sample shift to higher K, close to that of PtO_2, implying an extra Pt-O coordination under +0.15 V, likely arising from H_2O adsorption on Pt sites. Therefore, the WT results are highly consistent with the EXAFS fitting results.

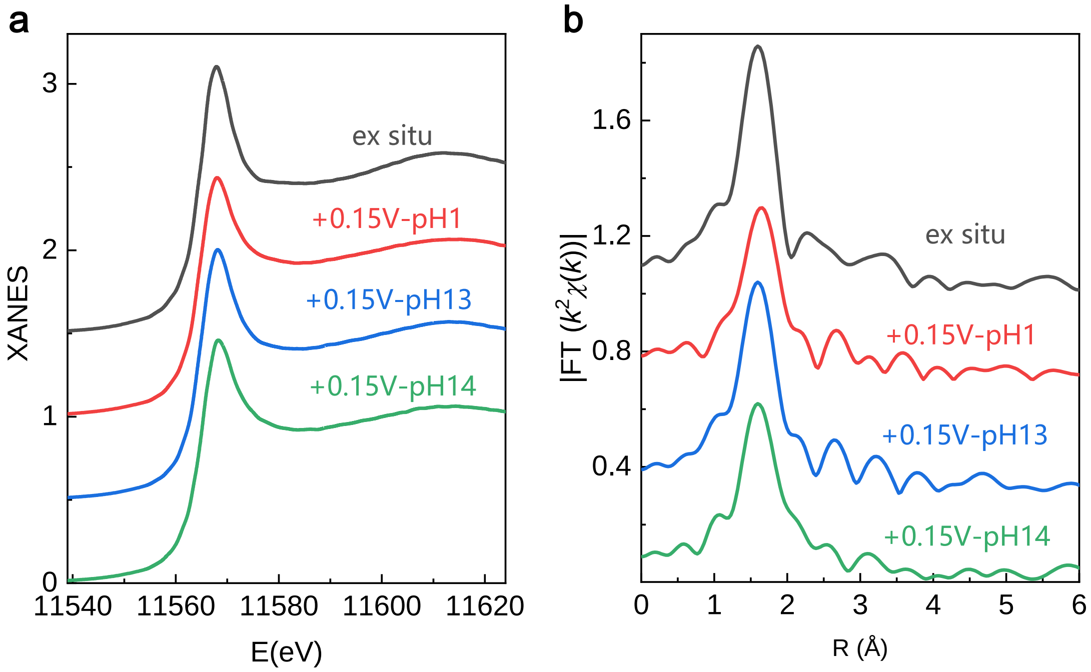

Supplementary Figure 18. XANES (a), and EXAFS (b) spectra of the ex-situ sample and under +0.15V (versus RHE) in wide-pH electrolytes. The intensities of the white-line peak and FT peak are also substantially reduced under +0.15V in pH1 (0.1 M $HClO_4$) and pH14 (1.0 M NaOH), akin to that in pH13 (0.1 M NaOH). These unanimous variations in the wide-pH electrolytes confirming the universality of the near-free-state Pt single atomic sites, leading to consistently excellent HER performance both in alkaline and acid.

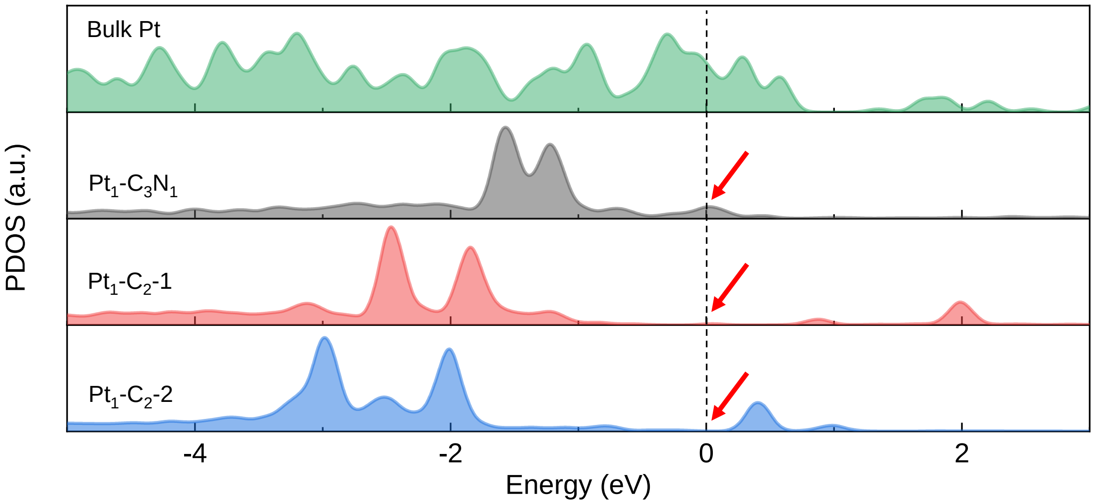

Supplementary Figure 19. Calculated partial density of states (PDOS) of Pt 5*d* orbitals for Pt_1-C_3N_1, Pt_1-C_2 and bulk Pt. The red arrows are pointing at the peaks near the Fermi level (0 eV), where the stronger peak of Pt_1-C_3N_1 suggests stronger hybridization between Pt and C/N orbitals than those of Pt_1-C_2-1 and Pt_1-C_2-2.

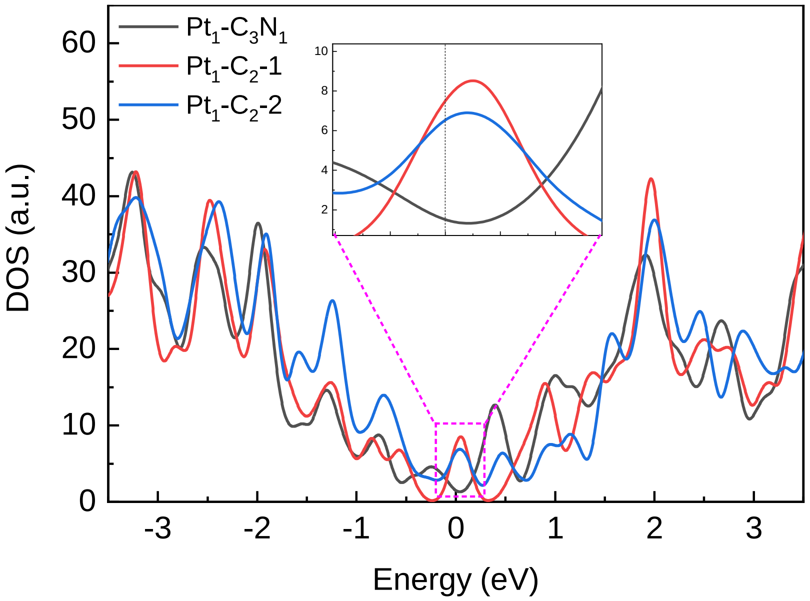

Supplementary Figure 20. Calculated DOS of Pt_1-C_3N_1 and Pt_1-C_2 systems. Different from the DOS of in Fig. 3b, this include contributions from N and C in the substrate, thus the peak near the Fermi level (0 eV) reflect the activity of the structure interacting with H_2O and H species. As shown in the inset, compared to Pt_1-C_3N_1, the prominent peaks of Pt_1-C_2 surfaces near the Fermi level imply higher localized electron density on Pt site, which may benefit the activation of H_2O molecules and thus boosts HER activity.

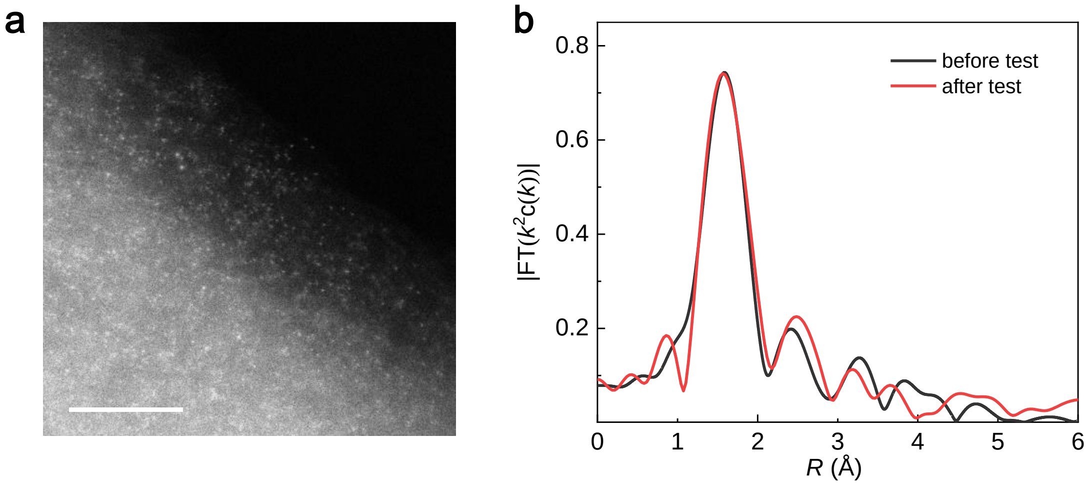

Supplementary Figure 21. HAADF image (a) and XAFS spectra (b) of the Pt_1/N-C catalyst after the stability test in 1.0 M KOH. Length of scale bar is 5 nm. The catalyst was sonicated and dispersed in ethanol from the electrode after test, then the ink was used for STEM measurement. It can be discerned that the single atomic state and monodispersion of Pt were preserved after 20-hours durability test.

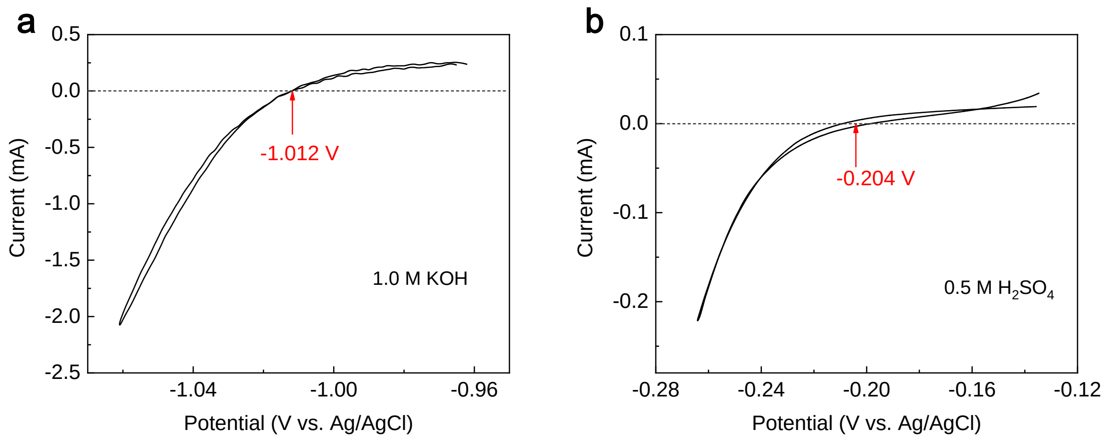

Supplementary Figure 22. Potential calibration of the Ag/AgCl reference electrode in high purity H_2-saturated 1.0 M KOH (a) and 0.5 M H_2SO_4 (b). Pt mesh was used as working electrode and CV was run at a scan rate of 1 mV s^{-1}. Indicated by the red arrows, the average of the two potentials at the current of zero was considered as the thermodynamic potential for the hydrogen reaction, i.e. 0 V versus reversible hydrogen electrode.

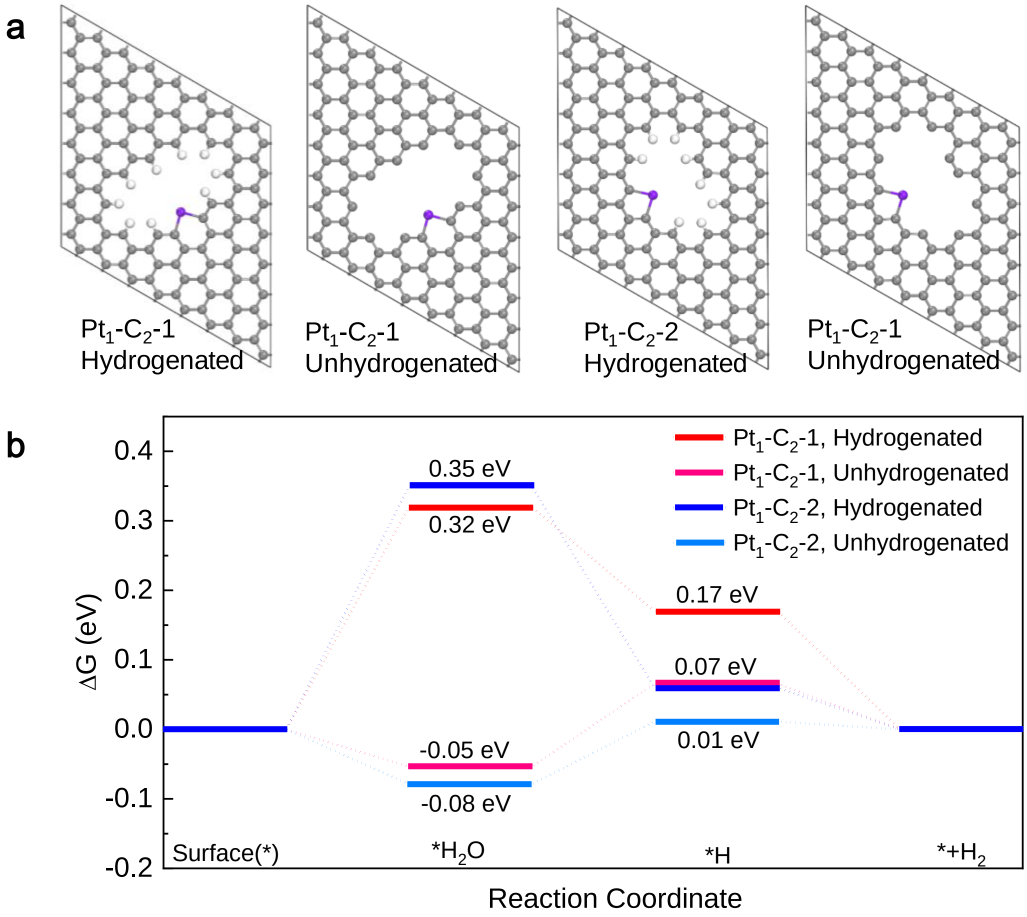

Supplementary Figure 23. (a) The DFT models of hydrogenated and unhydrogenated Pt_1-C_2 structures. (b) The corresponding H_2O and H adsorption energies. It is revealed that both water and hydrogen absorption are improved when the carbon dangling bonds are unsaturated by hydrogen atoms in the defects. During electrochemical reduction in proton-rich electrolytes, the carbon atoms with dangling bonds could be spontaneously hydrogenated by the protons. Therefore, we employed the fully hydrogenated models in the main text.

Supplementary Tables

Supplementary Table 1. Structural EXAFS curve-fitting parameters using the ARTEMIS module of IFEFFIT (The underlined parameters are fixed during fitting).

<table>
<tr><th>Sample</th><th>Path</th><th>N</th><th>R (Å)</th><th>σ^2 (10^{-3}Å^2)</th><th>ΔE_0 (eV)</th><th>R-factor</th></tr>
<tr><td rowspan="2">Ex-situ</td><td>Pt-N</td><td>1.0±0.2</td><td>2.09±0.02</td><td rowspan="2">3.6±1.2</td><td>7.1±1.0</td><td rowspan="2">0.007</td></tr>
<tr><td>Pt-C</td><td>3.2±0.1</td><td>2.04±0.02</td><td>7.1±1.0</td></tr>
<tr><td rowspan="2">+0.50 V</td><td>Pt-N</td><td>1.0±0.1</td><td>2.10±0.02</td><td rowspan="2">3.4±1.6</td><td><u>7.1</u></td><td rowspan="2">0.007</td></tr>
<tr><td>Pt-C</td><td>3.2±0.2</td><td>2.04±0.02</td><td><u>7.1</u></td></tr>
<tr><td rowspan="2">+0.15 V
Case 1</td><td>Pt-C</td><td>2.2±0.1</td><td>2.03±0.02</td><td rowspan="2">3.8±1.4</td><td><u>7.1</u></td><td rowspan="2">0.009</td></tr>
<tr><td>Pt-O</td><td>1.1±0.1</td><td>2.06±0.03</td><td>9.2±0.5</td></tr>
<tr><td rowspan="3">+0.15 V
Case 2</td><td>Pt-C</td><td>1.0±0.1</td><td>2.03±0.02</td><td rowspan="3">3.9±1.2</td><td><u>7.1</u></td><td rowspan="3">0.009</td></tr>
<tr><td>Pt-N</td><td>1.1±0.1</td><td>2.06±0.02</td><td><u>7.1</u></td></tr>
<tr><td>Pt-O</td><td>1.1±0.1</td><td>2.08±0.03</td><td>9.2±0.5</td></tr>
<tr><td>-0.07 V
Case 1</td><td>Pt-C</td><td>2.2±0.1</td><td>2.07±0.02</td><td>4.0±1.2</td><td><u>7.1</u></td><td>0.007</td></tr>
<tr><td rowspan="2">-0.07 V
Case 2</td><td>Pt-C</td><td>1.0±0.1</td><td>2.04±0.02</td><td rowspan="2">4.2±1.2</td><td><u>7.1</u></td><td rowspan="2">0.009</td></tr>
<tr><td>Pt-N</td><td>1.1±0.1</td><td>2.07±0.02</td><td><u>7.1</u></td></tr>
<tr><td>+0.15 V-pH1</td><td>Pt-C</td><td>2.3±0.1</td><td>2.06±0.02</td><td>4.1±1.3</td><td><u>7.1</u></td><td>0.009</td></tr>
<tr><td rowspan="2">+0.15 V-
pH14</td><td>Pt-C</td><td>2.2±0.1</td><td>2.02±0.02</td><td rowspan="2">4.5±1.2</td><td><u>7.1</u></td><td rowspan="2">0.007</td></tr>
<tr><td>Pt-O</td><td>1.1±0.2</td><td>2.08±0.03</td><td>9.4±0.8</td></tr>
</table>

Supplementary Table 2. Bader charge analysis of the Pt-standard samples, the ex-situ samples and the samples during reaction. Actual charge of Pt and Bader charge of Pt, N, C, O and H.

Sample	Actual charge (*e*)	Bader charge (*e*)				
	Pt	Pt	N	C	O	H
$PtCl_2$	9.48	+0.52	-	-	-	-
$PtCl_4$	9.08	+0.92				
Pt_1-C_1N_3	9.36	+0.64	-1.19<br>-1.15<br>-1.19	-0.09	-	-
Pt_1-C_2N_2	9.41	+0.59	-1.16<br>-1.16	-0.16<br>+0.11	-	-
Pt_1-C_3N_1	9.53	+0.47	-1.23	-0.17<br>-0.01<br>-0.04	-	-
Pt_1-C_2-1	9.89	+0.11	-	-0.05<br>-0.05	-	-
Pt_1-C_1N_1-1	9.77	+0.23	-1.55	-0.09	-	-
Pt_1-C_2-2	9.88	+0.12	-	-0.12<br>-0.14	-	-
Pt_1-C_1N_1-2	9.90	+0.10	-1.14	-0.12	-	-
H_2O-Pt_1-C_3N_1	9.56	+0.44	-1.13	-0.13<br>-0.11<br>-0.19	-1.24	+0.57<br>+0.61
H_2O-Pt_1-C_2-1	9.89	+0.11	-	-0.25<br>-0.26	-1.23	+0.47<br>+0.60
H_2O-Pt_1-C_1N_1-1	9.90	+0.10	-1.08	-0.05	-1.18	+0.55<br>+0.58

H_2O-Pt_1-C_2-2	9.88	+0.12	-	-0.12<br>-0.14	-1.24	+0.63<br>+0.56
H_2O-Pt_1-C_1N_1-2	9.93	+0.07	-1.16	-0.03	-1.20	+0.55<br>+0.59
H-Pt_1-C_3N_1	9.44	+0.56	-1.22	-0.04<br>-0.12<br>-0.18	-	+0.10
H-Pt_1-C_2-1	9.88	+0.12	-	+0.07<br>+0.06	-	-0.26
H-Pt_1-C_1N_1-1	9.86	+0.14	-1.15	-0.08	-	-0.18
H-Pt_1-C_2-2	9.84	+0.16	-	-0.12<br>-0.03	-	-0.27
H-Pt_1-C_1N_1-2	9.82	+0.18	-1.23	-0.07	-	-0.15

Supplementary Table 3. The HER activity of the Pt_1/N-C compared with other recently reported single-atom catalysts in acid and alkaline.

Catalysts	Mass loading (mg/cm^2)	Overpotential (mV)		References
		acid	alkaline	
Pt_1/N-C	0.25	19	46	Our work
$Pt_{SA}/Mo_2TiC_2T_x$	1.0	30	-	Ref. 1
Fe/GD	-	66	-	Ref. 2
Pt/np-$Co_{0.85}Se$	2.04	58	58	Ref. 3
Ru-NC	0.20	29	12	Ref. 4
Pt-GT-1	0.28	18	-	Ref. 5

Supplementary Notes

Supplementary Note 1. The turnover frequency (TOF) per Pt site of the Pt_1/N-C was calculated according to the following equation:

$$\mathrm{TOF} = |J| / (2nF)$$

Where J is the current density (A cm^{-2}) from the LSV measurement, F is the Faraday constant (C mol^{-1}), n is the number of active sites per geometric area (mol cm^{-2}). The factor 1/2 results from the fact that two electrons are required for one hydrogen molecule. The Pt content of Pt_1/N-C determined by the ICP-OES is ca. 2.5 wt%, and mass loading on the electrode is ~0.25 mg cm^{-2}. Thus, n is calculated as:

$$n_{Pt_1/N\text{-}C} = \frac{2.5\,\% \times 0.25\,\mathrm{mg/cm^2}}{195.084\,\mathrm{g/mol}} = 3.2 \times 10^{-8}\ \mathrm{mol/cm^2}$$

$$n_{Pt/C} = \frac{20\,\% \times 0.25\,\mathrm{mg/cm^2}}{195.084\,\mathrm{g/mol}} = 25.6 \times 10^{-8}\ \mathrm{mol/cm^2}$$

Supplementary Note 2. The oxidation states of Pt in the Pt_1-C_3N_1, Pt_1-C_2N_2 and Pt_1-C_1N_3 samples are calculated based on the bader charge analysis. Bader charge of bulk Pt, $PtCl_2$ and $PtCl_4$ are linearly fitted with their oxidation states, which are known as 0, +2 and +4, respectively. As shown in Fig. S24 below, the oxidation states of Pt_1-C_3N_1, Pt_1-C_2N_2 and Pt_1-C_1N_3 turn out to be 1.96, 2.48 and 2.70, respectively, obtained by linear interpolation from the fitting line.

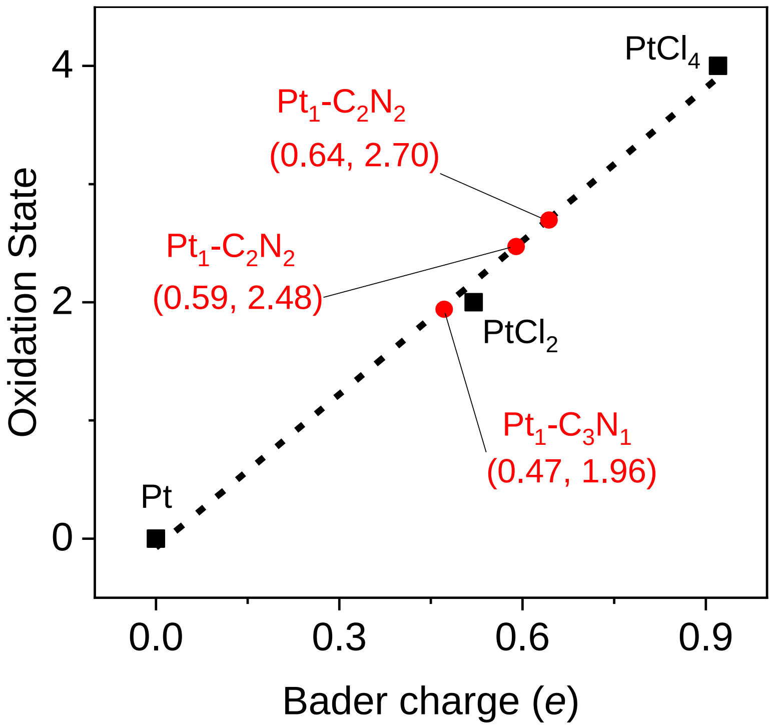

Supplementary Figure 24. The bader charge and fitted oxidation states of Pt_1-C_3N_1, Pt_1-C_2N_2 and Pt_1-C_1N_3 samples.

Supplementary Note 3. The HER mechanisms are generally accepted as:

Volmer step:

$H^+ + M + e^- \rightleftarrows M\text{-}H^*$ (in acid)

$H_2O + M + e^- \rightleftarrows M\text{-}H^* + OH^-$ (in alkaline)

Followed by Tafel step:

$2M\text{-}H^* \rightleftarrows 2M + H_2$ (in both acid and alkaline)

or Heyrovsky step:

$M\text{-}H^* + H^+ + e^- \rightleftarrows M + H_2$ (in acid)

$M\text{-}H^* + H_2O + e^- \rightleftarrows M + H_2 + OH^-$ (in alkaline)

The HER pathway in alkaline initiates with H_2O adsorption, followed by electrochemical reduction of adsorbed H_2O into adsorbed H atom (H*) for the next H_2 generation. Different from the case in acid, H_2O adsorption makes a substantial impact on the HER activity in alkaline[6,7]. Thus, the bonding energies of H_2O and H on Pt_1/N-C surface were examined to find the origin of HER activity in both alkaline and acid solutions. The Pt_1-C_3N_1, Pt_1-C_2 and Pt_1-C_1N_1 surfaces were constructed, for the purpose of simulating the configuration variations of Pt site under ex-situ and working conditions, and a illustrative catalytic cycle for alkaline HER is proposed based on the Pt_1-C_2 configuration as an example, shown in Fig. S25 below. It was revealed that the H_2O adsorption is more preferable on the Pt_1-C_2 than that on the Pt_1-C_3N_1. Also, the H adsorption becomes more moderate. Therefore, we can conclude that the evolved near-free state of Pt is the origin of the excellent HER activity.

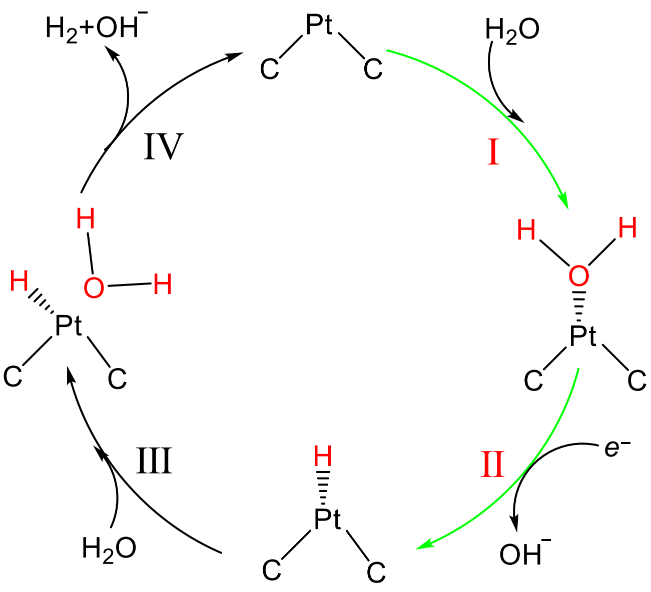

Supplementary Figure 25. Illustrative example of alkaline HER mechanism on Pt_1-C_2. The catalytic cycle is initiated by adsorption of H_2O onto Pt (step I), the absorbed H_2O then dissociated into absorbed H (H*) on Pt and OH^- (step II), followed by another proton from an adjacent H_2O molecule reacting with the first H* to generate H_2 (steps III–V).

Supplementary Note 4. Bader charge analysis were used to estimate the valence state of the Pt-standard sample. The bader space is divided into regions by surfaces that run through minima in the charge density, without gradient of the electron density normal to the surface. Compared to the basis set sensitive Mulliken analysis with arbitrary charge assignment, bader charge was more suitable as it is based solely on the charge density, it can also be used to analyze plane wave based calculations as well as calculations using atomic basis functions.[8]

Supplementary Note 5. The free energy of each intermediate species can be presented as:

$$G = E_{DFT} + E_{ZPE} + \int C_p dT - TS$$

Where E_{DFT}, E_{ZPE} and S denote as the ground state energy, zero point energy and entropy respectively. We set the temperature T as 298.15K and calculate E_{ZPE} and S by means of vibrational frequencies calculations. We performed harmonic approximation and neglected the slab contribution. The adsorption free energy can therefore calculated as:

$$\Delta G_{ads} = \Delta E_{ads} + \Delta E_{ZPE} - T\Delta S$$

ΔE_{ads} is the adsorption energy, ΔE_{ZPE} and ΔS are the difference of zero point energy and difference of entropy, respectively. The solvent effects were considered by using the Poisson-Boltzmann implicit solvent model[9] with the dielectric constant set to be 78.4 for solvent water.

Supplementary References

1. Zhang, J. *et al.* Single platinum atoms immobilized on an MXene as an efficient catalyst for the hydrogen evolution reaction. *Nat. Catal.* **1**, 985-992 (2018).
2. Xue, Y. *et al.* Anchoring zero valence single atoms of nickel and iron on graphdiyne for hydrogen evolution. *Nat. Commun.* **9**, 1460 (2018).
3. Jiang, K. *et al.* Single platinum atoms embedded in nanoporous cobalt selenide as electrocatalyst for accelerating hydrogen evolution reaction. *Nat. Commun.* **10**, 1743 (2019).
4. Lu, B. *et al.* Ruthenium atomically dispersed in carbon outperforms platinum toward hydrogen evolution in alkaline media. *Nat. Commun.* **10**, 631 (2019).
5. Tiwari, J. N. *et al.* Multicomponent electrocatalyst with ultralow Pt loading and high hydrogen evolution activity. *Nat. Energy* **3**, 773-782 (2018).
6. Strmcnik, D. *et al.* Improving the hydrogen oxidation reaction rate by promotion of hydroxyl adsorption. *Nat. Chem.* **5**, 300-306 (2013).
7. Subbaraman, R. *et al.* Enhancing hydrogen evolution activity in water splitting by tailoring Li^+-$Ni(OH)_2$-Pt interfaces. *Science* **334**, 1256-1260 (2011).
8. Henkelman, G., Arnaldsson, A. & Jonsson, H. A fast and robust algorithm for Bader decomposition of charge density. *Comp. Mater. Sci.* **36**, 354-360 (2006).
9. Mathew, K., Sundararaman, R., Letchworth-Weaver, K., Arias, T. A. & Hennig, R. G. Implicit solvation model for density-functional study of nanocrystal surfaces and reaction pathways. *J. Chem. Phys.* **140**, 084106 (2014).
